# People are STRANGE: towards a philosophical archaeology of self

**DOI:** 10.1007/s11097-024-10002-1

**Published:** 2024-07-04

**Authors:** Lambros Malafouris

**Affiliations:** Cognitive and Anthropological Archaeology, Institute of Archaeology, University of Oxford, 36 Beaumont Str, Oxford, OX1 2PG UK

**Keywords:** Situated person perspective, Self-bounding, Material engagement, Agency, Body ownership, Enactive in/dividuation, Tool use, Personal ornamentation

## Abstract

Philosophical preoccupation with the hard problem of self-consciousness often takes human becoming for granted. In archaeology, the opposite is the norm. The emphasis is on when and how we became human while the problem of self (how did the ability to think about one’s own self come about? ) is largely neglected. This article suggest that those two aspects of human becoming cannot be meaningfully disentangled: humans are both persons and members of a species. I argue that people are STRANGE. I use the acronym STRANGE to describe the Situated TRANsactional and GEnerative process by which the human species (nature) and the human self (culture) become co-constituted in the lived space of material engagement. I propose that to study this middle space of self-becoming a synergy of enactive and situated perspectives from philosophy and archaeology is needed. Drawing on material engagement theory I sketch out my vision of what this synergy entails based on the notions of self-bounding, enactive in/dividuation, and the situated person perspective. I use the archaeological examples of stone knapping (toolmaking) and early body ornamentation to substantiate some of the main issues and methodological challenges.

## Introducing the problem

Think of the sentient body of an early stone knapper from the Olduvai Gorge in Africa preparing a sharp-edged chopping tool some 2.5 million years ago (Semaw et al., 1997), or to take a different and more recent example, imagine the dwellers of the Blombos Cave in South Africa intentionally decorating their bodies using shell beads at c.75 kya (Henshilwood et al., 2004; d’Errico et al., 2005). What kind of self-experience should we ascribe to them? One could hypothesize, based on comparative findings from other animal and developmental psychology, that the possession of a minimal subjectivity must have been already present at an early stage in human evolution. But exactly when, and through what processes, did human self-consciousness (awareness of experiencing and of being the experiencer) emerge? The making of human self is, arguably, among the most fundamental issues of human becoming. It also provides fertile ground for cross-disciplinary collaboration between philosophy and archaeology.

Take the case of the Blombos Cave. As mentioned, some of the earliest examples of deliberate engraved ochre and perforated shell beads used for personal decoration have been excavated there (Henshilwood et al., 2009; Henshilwood & Dubreuil, 2011). The practice of bodily decoration indicates ability to become the object of one’s own reflection, and to acquire the perspective of the other (second person perspective). Still, we have no way to know how the inhabitants of this cave would have reacted to the view of their face and body as seen on the surface of the mirror. Would they have smiled or shown embarrassment, or is it maybe, that confronted with their specular image, our African ancestors at Blombos would have become paralyzed of terror and anguish, “covering their mouths and hiding their heads”, as reported by the anthropologist Edmund Carpenter for the Biami tribe in Papua New Guinea (Carpenter, 1975, 452–453; Koukouti & Malafouris, 2020 ch. 2). In other words, would the people dwelling at Blombos have been capable of explicit self-recognition? Should we think of them as self-conscious beings capable of reflecting upon one’s own past, present, and future?

These questions, of course, may seem difficult, if not impossible, for archaeologists to address in an entirely satisfactory manner. Archaeology, apparently, lacks any ready-made methodological substitute for the classical ‘mirror self-recognition task’ (Gallup, 1970, 1979; Rochat & Zahavi, 2011; Lobaccaro & Bacaro, 2021), or for the interview methods and assessment tools adopted in the phenomenological psychopathology of self (Fuchs, 2008, 2017; Fuchs & Röhricht, 2017; Parnas et al., 2005; Sass, 2010, 2014; Stanghellini et al., 2019; Gallagher, 2024). From an archaeological perspective, we have no direct way to test for self-identification. What we do have nonetheless, is a rich material record that, combined with anthropological and experimental (actualistic) studies (Clarkson & Shipton, 2015; Nonaka et al., 2010; Wadley, 2010; Pargeter et al., 2019, 2020; Stout & Chaminade, 2007; Stout et al., 2008; Walls, 2016, 2019; Walls & Malafouris, 2016), potentially allows us to study the multitemporal dynamics, patterns and traces of self-becoming. Naturally, the question of self-consciousness is not one that can be easily extrapolated from the archaeological record without the appropriate theoretical framework. The material remains of the past may speak in their own peculiar enactive semiotic idiom (Malafouris, 2008a, b, c, 2013, 2021c), but they lack any direct equivalent of ‘mineness’ (Zahavi, 2005). First person experiences and pronouns like ‘me’ and ‘I’ do not fossilize nor do they leave any readily identifiable and universal material signature.

What is it then that we can hope to achieve by studying self-becoming in human prehistory? What can we contribute to the question of self-consciousness on the basis of archaeological evidence? I will endeavour to show that many developmental and ontological aspects of the paradox of human subjectivity^1^ can be somewhat illuminated and partially resolved from a material engagement perspective (Malafouris forthcoming). Maybe more important than actually resolving the archaeological conundrum of self is to show that it can be avoided no more. It cannot be avoided because it is always present, underlying every aspect of human evolution and our perception of the archaeological record. To explain: whether archaeology explicitly looks for the self or not, the existence of a transparent phenomenal inner subjectivity, moulded on the prototype of the modern Western individual, is usually assumed before and behind even the earliest human intentional actions and behavioural traces. But whence does this confidence concerning the universality of a similar sense of ‘me’ and ‘mine’ emanate? Can we simply presume the access to an objectified self in the past? I think not.

Certainly, the evidence of stone knapping from the early archaeological record can be seen as indexes of an acting body. But do they also provide, *in themselves*, direct evidence of a self-aware acting body? Or is it that the acting body and what we call self or person are the same? We often think of the human body as the locus of self. That is, we think that we are a body and that we have a body. Many researchers would prefer to see the self as something located ‘within’ that body. Others would prefer to describe this intimate association between the self and the body (especially the brain) as an identity. I will argue below that the topology of this close relationship between self and the ‘lived’ body, as well as the mereology (from the Greek *meros* ‘part’)^2^ of its component parts is more plastic and reconfigurable than we might have thought.

Useful to note in this connection is that our taken for granted idea of the delimited ‘individual’ has been subjected to fierce criticism in anthropology. The anthropology of self testifies that for many communities in the world persons are not necessarily predicated either on the category of individual self-contained being or even on the category human. Marilyn Strathern’s discussion of the ‘dividual’ Melanesian person in her much quoted work *The Gender of the Gift* offers the classical example of such a context (Strathern, 1988; Ramsey, 2000)^3^. Her exposition of the Melanesian person as a “composite site” where aspects of the self are distributed among others, as are others in oneself, offers a useful alternative lens for looking at pre-modern subjectivities. There are many more ethnographies that support a perspectival view of selfhood based on relatedness. From Nurit Bird-David’s interpretation of the Nayaka of South India (Bird‐David 1999), to Rane Willerslev’s account of the Siberian Yukaghirs (Willerslev, 2007), to the work of Eduardo Viveiros de Castro amongst Amerindian peoples in Amazonia (1998) and more recently of Fernando Santos-Granero’s (2012) among the Yanesha of eastern Peru, anthropological studies of personhood clearly support the authority of intersubjective compound beings over the isolated individual person. As Sahlins remarks: “the being-ness of humans is not confined to singular persons” (2011, 227). Understanding what this permeable relationally constituted self means demands that we move a step further than merely recognizing the undisputable anthropological fact that conceptualizations of self vary cross-culturally, often quite sharply. It no longer suffices to claim, like Clifford Geertz did, that for Java, Bali, and Morocco, the idea of selfhood “differs markedly not only from our own but, no less dramatically and no less instructively, from one to the other” (Geertz, 1975, 48 − 9). We must move beyond issues of ‘self-representation’ and instead, strive to understand the conditions of human *situatedness* and the varieties and possibilities of self-becoming that they allow, recognising that the process of human individuation is not confined to the boundaries of the individual.

Archaeology, anthropology, philosophy and cognitive science, working together, should be concerned, on the one hand, with the ontological standing of those relational, perspectival and ‘non-modern’ conceptualizations of self, and on the other, with the possible contribution that such understandings can make to the ways we traditionally conceptualise the nature and boundaries of human self. Our modernist predilection for ‘internalist’ ontologies that situate selfhood ‘within’ the individual should not blind us to the possibility that before this ‘me’ of which the origins we seek in the past, can be a ‘we’ or a ‘many’. I mean that not in the conventional intersubjective sense but in a more radical transactional, suprapersonal sense where self-boundaries are changeable and extendable to the outside world, rather than fixed at the surface of the skin. It is these possibilities that I wish to bring forth and explore in this paper, adopting a material engagement perspective and advocating for a synergy between enactive-ecological philosophy and cognitive archaeology.

Three major kinds of questions could form the basis of our analytical efforts, i.e., epistemological, ontological, and comparative:

Epistemological questions are the ones which probably raise the biggest challenge: How can we study the self in the past? Which features of the archaeological record might provide access to what aspects of self-experience? Take the example of stone tools, or any of the more recent behavioural traits (e.g. burial, ornamentation, blade technologies, hearth construction, evidence of exchange networks, organized use of domestic space) associated with the emergence of what is usually referred to as ‘modern’ human intelligence or behavioural modernity (d’Errico & Stringer, 2011; Henshilwood & Marean, 2003; McBrearty & Brooks, 2000). Does self-consciousness leave any distinctive material and behavioural traces?

The epistemological questions just described are depended upon another set of even more basic ontological queries regarding the meaning and constitution of self: what is it that we call self? What is it made of? What are the main processes implicated in the emergence of self-awareness? Which of these processes can be seen as integral or constitutive parts of the ability to become self-aware (e.g., sense of agency, ownership, autobiographical memory, self-talk, mental time travel), and which might be viewed mere consequences, or by-products, of this ability for self-reflection (e.g., self-recognition, Theory-of-Mind)? When, why, and how did humans develop a sense of ownership and start to think of themselves as the authors of their actions (sense of agency)?

This brings us to the third major category of comparative questions: What, if anything, is unique about human self-awareness? What is the meaning of the word self in animal self-recognition? Can it be argued that other animals are self–aware? Or is it that talk of self-consciousness in other species simply represents another instance of our anthropocentric bias towards “animalizing humans” and “humanizing animals”, what the neuroscientist Raymond Tallis calls the “fallacy of misplaced explicitness” (2011, 159). Although a basic proto-self can be seen as the emergent by-product of certain species, in the case of humans the same basic processes seem to give rise into a radically different kind of self-reflective ontology unlike anything we see in other animals. What makes human self awareness so different then? Some animals seem to present a number of features indicative of a core proto-self system (Gallup, 1970;1979; Damasio, 1999). But they never make the passage to the reflective, or conceptual stages of selfhood. A nut-cracking chimpanzee can certainly effect a forceful stroke, or potentially succeed in the mirror self-recognition task, but most probably lacks any awareness of agency or a true understanding of causality (for review of the debate see Visalberghi & Tomasello, 1998; Penn & Povinelli, 2007*)*. Why is that? How did humans move beyond this basic level of the phenomenal proto-self? Can there be a self without or prior to language? At what level or stage in the development of human self-consciousness is language required?

Before I begin addressing some of these questions, it is important to make explicit some of the main assumptions that needs overcome as well as the theoretical framework that I will use to tackle them.

## Overcoming misconceptions and discontinuities

There are two common misconceptions or discontinuities in the way some of the issues and questions we discussed above have been traditionally approached and which, I suggest, we should try overcome.

One misconception concerns our deeply entrenched modernist presuppositions about the ontological boundaries of self. It concerns the classical distinction between ‘inside’ and ‘outside’. We naturally come to think that we have an inner self as a matter of unchallengeable fact^4^. But this strong *internalist* tendency to define and locate the self ‘within’, rather than ‘outside’ us, what Charles Taylor calls a sense of “inwardness”, is in large part a historical property of our modern thinking (1989, 111)^5^. This *internalist* tendency often blind us to any alternative suprapersonal situated perspectives. As mentioned above, one strategy to overcome this problem is to look at the anthropological debates over the general idea of the individual: dividual distinction (e.g. Busby, 1997; Strathern, 1988; LiPuma, 1998; Smith, 2012; Fowler, 2016). Can ethnography, philosophy, and cognitive science produce commensurable sources of evidence on the question of self? Although, for many decades now, there has been considerable interest in such integration a comprehensive attempt at answering that question has never been attempted (for a review of the problems see Bloch, 2012, ch. 6; Smith, 2012). Needless to say, the use and possible contribution of comparative ethnography to philosophical archaeology is to help us in making informed interpretations about self in the past and not to impose ill-considered anachronistic analogies (Fowler, 2003, 2016; Malafouris, 2023).

This brings us to the another major misconception which we need to overcome. It concerns the commonly perceived discontinuity between the minimal or basic (pre-reflective) dimensions of self and the conceptual, reflective or socio-cultural attributes of selfhood. The former, i.e., minimal self, is generally been conceived as the product of human biology and evolution, whereas the latter, i.e., conceptual self, the product of enculturation and personal ‘narrative’ (for a discussion of the distinction between ‘minimal’ and ‘narrative’ self, see Gallagher, 2000, 2013, 2024). On this construal, the physical minimal self is amendable to scientific analysis and generalization, whereas the narrative self can only be the object of narrative analysis and ethnographic description. At one level, such a division of labour is convenient since it provides the purified epistemological space that enables, on the one hand, neuroscience to focus, for example, on the role of the right temporoparietal junction (rTPJ) in human embodiment and sense of agency (Tsakiris et al., 2008), and on the other hand archaeology and anthropology to focus on the comparative analysis and interpretation of cultural differences in the experience and concept of self. However, it can also be argued that this epistemic divide is misleading on two counts: for one thing, it implies the existence of two ontologically different and epistemologically isolated layers of self-experience that can exist or studied independently of one another. On the other hand, it reiterates a problematic assumption about the priority of the minimal, pre-reflective self over the reflective or narrative self. The implicit claim is, in short, that whether one thinks in terms of human evolution, ontogeny, or psychopathology, only the minimal self really matters. Cultural differences in self-concept have significance (and thus reality) only if they can be shown, for instance by way of brain imaging, that they influence or change the underlying neural substrates of self representation (e.g. Zhu et al., 2007; Zhang et al., 2006). This separatist logic of discontinuity among synaptic, bodily, social aspects of self is also implicit in many anthropological studies where the self is recognized as a cultural construct and is treated as an abstract symbolic representation, largely separated from the minimal bodily aspects of self-consciousness. What this logic implies (wrongly in my view) is that the situated objectifications of the experience of agency or ‘mineness’ are arguably less sensitive – if not totally immune – to cross-cultural variations, hence potentially less interesting from an anthropological perspective.

In this connection, a central guiding assumption for philosophical archaeology needs to be made clear: self makes little sense as an isolated property of the human brain. All major self-related processes are inseparable from, although not necessarily identical with, a lived body which is by definition a situated body (co-constituted and embedded in a specific material environment). It is one thing to say that, for instance, certain areas of human brain, like the insular cortex, the anterior cingulate cortex, or the medial prefrontal cortex play a special role in the creation of the self. It is indeed another, quite different thing to say that the self ‘resides’ in any of these areas of the brain. The first seems to be at present a well-supported neuroscientific finding (e.g. Christoff, et al. 2011; Philippi et al., 2012; Vogeley & Fink, 2003; Legrand & Ruby, 2009). The second is a good example of how a valuable finding can be turned into a category mistake, as it is often the case with questions of ‘localization’ – that is, questions about ‘where in the brain is the self?’ (Vogelely & Gallagher 2011; Gillihan & Farah, 2005). The claim I put forward is not simply that self processes are more likely to emerge from distributed interactions among networks of brain regions. Rather, the central thesis of my approach, which is grounded on the principles of material engagement theory (MET) (Malafouris, 2013, 2019), is that *self is more than a brain: it is an ongoing process (becoming) bound up with a situated lived body and its changing socio-material environment*. The central assumption is that the material world often becomes a constitutive part of the human self-system both from an ontogenetic and phylogenetic perspective. From a material engagement perspective, it would be wrong to conceive of the different gradations and types of self-consciousness (minimal or narrative) as lacking a material substrate (outside the brain). Material culture provides a powerful means for grounding self in action across the scales of time. In that sense, studying human modes of engagement we can gain access into the different ways by which the basic feeling of one’s own bodily presence in the world can be transformed to objectified self-knowledge or acquire spatio-temporal extension and coherence. Emphasis should be placed on understanding the plasticity of self-boundaries and the relational processes of metaplasticity (Malafouris, 2008a,b; Malafouris, 2010a,b; 2015) by which those boundaries become constituted, rather than on interactions between static entities with pre-defined boundaries. The objective is to deliver an account of the self as an ongoing process (i.e., self-becoming) and to highlight the active role and redefine the meaning of material culture in that process. Obviously, there are strong intellectual ties here with theories of extended, distributed and enactive-ecological cognition (Clark, 2007, 2008; Hutchins, 2010; Thompson, 2007, 2014; Di Paolo, 2009; Gallagher, 2013, 2017, 2023a, 2024; Noë, 2009; Fuchs, 2017; Heersmink, 2020). Notions of extended or distributed selfhood have already been variously employed in archaeology and anthropology (Bateson, 1973; Gell, 1998; Fowler, 2002, 2003; Gosden, 2008; Malafouris, 2008a, b, c; Knappett & Malafouris, 2008; Malafouris & Renfrew, 2010; Malafouris & Koukouti, 2022) and offer a productive relational foundation to explore the different dimensions of self-related cognition in situated action.

The extended plasticity of self means that it cannot be characterized and understood simply, according to some internal, and biologically predetermined taxonomy of bodily phenomenal properties. This does not mean that invariable phenomenological patterns do not exist. It only means that any phenomenological description or explanation needs to take into consideration the specific socio-material parameters that operate beyond the skin in the lived (peripersonal and interpersonal) space. Self-becoming is participatory, reciprocal and prosthetic: not a becoming *of* but a becoming *with* and *through.* In other words, my thesis is that people are STRANGE.

## Selfbound and STRANGE

**Fig. 1:**
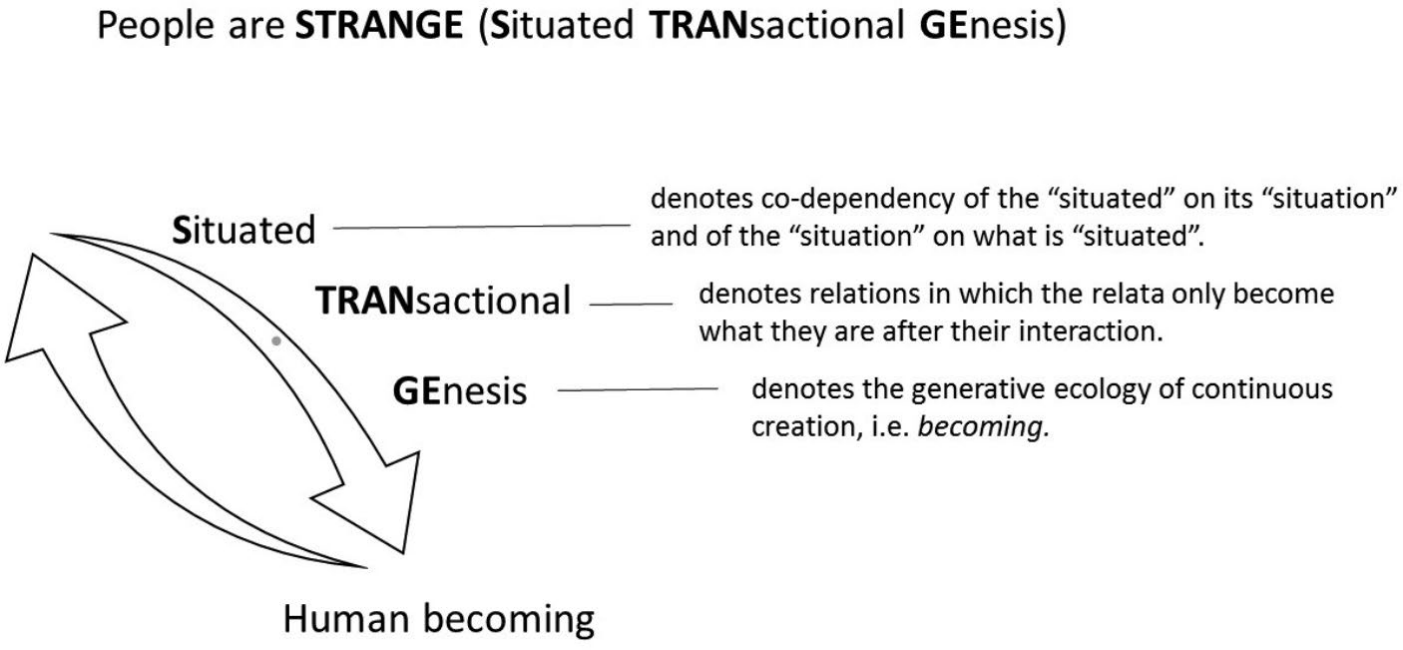
People are STRANGE (Situated TRANsactional GEnesis)

I use the acronym STRANGE to describe the process of Situated TRANsactional GEnesis by which self-becoming is realised (Malafouris, forthcoming) (Fig. 1). In particular:

*Situated* denotes human embeddedness and dependency to specific material environments (the situation). ‘Situatedness’ is not a mere positioning of a living occurrence according to a possible set of spatiotemporal coordinates. Situatedness designates something deeper: *the co-constitution of the situated and the situation*. This meaning of situatedness carries important ontological and epistemological implications. From an ontological point of view, it denotes the necessary unity between people and things (in the broadest sense of the term that includes both objects and material environments). From an epistemological point of view, it denotes the unity between the observing and the observed^6^. When we speak of human situatedness we should not be thinking of an environment ‘out there’ surrounding an individual body or person, but of a *hylonoetic* field (from Greek *hylē* for matter and *noêsis* for intelligence) where brains, bodies and things are entangled. For instance, when in the following section I describe the knapper (early toolmaker) as situated, I do not refer solely or primarily to the physical positioning of the knapper’s body ‘in’ space. What I describe is the diachronic entanglement between the toolmaker’s body and the affordances of stone. The meaning of ‘in’ here refers to the *withness* and *throughness* of the knapper’s *thinging* (thinking and feeling with and through the stone) (Malafouris, 2021a,b). Which brings us to the second constituent of human STRANGEness.

*Transactional* describes the type of interaction that is distinctive of what it means to be situated ‘in’ an environment. Specifically, a transactional interaction is one where the interacting entities or processes only become what they are *after* their interaction (Dewey & Bentley, 1949). That is, their actual meaning and boundaries (what they exclude and what they include) do not predate their entanglement. Transactional relations resemble gift exchanges (see also Malafouris & Koukouti, 2022); they are characterised by uncertainty, anticipation, reciprocity, inalienability and incompleteness. I see them as the primary characteristic of human becoming. If we accept these gift-like qualities of human becoming, then we probably need to consider the possibility that there is no such thing as a free, boundless person. The knapper and the stone tool are co-produced; they are the products of their transaction. This transactional logic also explains what I mean when I say that humans are *thingers*, or refer to the process thinking as *thinging.* There is no directionality from humans to things or from mind to matter. We think *with* and *through* things only because things think *through* and *with* us (using human muscles and brains). Thinking is a joint transactional accomplishment of the fusion of people and things. The fusion I talk about can be experienced and observed at different levels and temporal scales. Focusing on the production of a single flake, in a given point in time, we may perceive the engagement between the knapper’s skilled body and the flaking affordances of the stone as a basic interaction. However, as I will explain below (section 4.1), the resistance of the stone opens up to the toolmaker a new field of situational affordances that will allow the exploration, discovery and realisation of their mutual capacities for joint action. Transacting with the stone the toolmaker is not just producing flakes for cutting but also extending the boundaries and possibilities of self-designation^7^. People and things are jointly appropriated and educated to sustain or discover their relational affordances.

*‘Genesis’* is the final characteristic of this relational domain. It denotes, specifically, the generative ecology of *continuous creation*, i.e. *becoming*. It refers to the perpetual generation and transformation of human self, i.e. *self-becoming.* Humans are creative organisms of a relational kind and prosthetic disposition, constantly changing their material environments opening up new paths of development (macro- and micro-genesis) and possibilities of self-becoming. This process of anthropogenesis dynamically combines phylogenesis (evolution), ontogenesis (development), and epigenesis (non-genetic inheritance processes). Relevant in this connection is the concept of ‘epiphylogenesis’^8^, introduced by the French philosopher of technics and technology, Bernard Stiegler, in order to distinguish technical evolution from biological evolution (phylogenesis). Stiegler’s theory of anthropotechnical evolution is also based on the assumption that what makes humans ‘human’ (species and person) is constituted by technics and it cannot be understood without them, that is, without understanding the material conditions of human becoming. More than the products of natural selection, humans have been evolving by means of creative material engagement (creative *thinging)*, constantly changing their material environments opening up new paths of development and possibilities of self-becoming (Malafouris, 2014, 2020, 2021b). Humans create things, and things in turn create us. Creative *thinging* (the discovery of new material signs and modes of enactive signification) both sets and expands the limits of human consciousness (Malafouris, 2014). Through the process of creative material engagement we transform the ways we touch and are being touched and make sense of others and the world. Our cores and peripheries, our insides and outsides; the very boundaries of the self: it all changes. New modes of engagement create new possibilities for self-observation and self-identification. Early human engagement with stone, to use our example of knapping, not only created new tools—in the functional sense of bodily extensions and ‘extrasomatic means of adaptation’ (Binford, 1972)—but also, a novel domain of self-making. The edge of stone was never just for cutting meat—we have been using that edge to change the affordances of the world and of our bodies, redirecting and redistributing the flows of energy and matter. Our attempt to develop a cognitive archaeology of human self-consciousness should start with the recognition of our self-bounding relationship with the material world.

Before I explain the notion of self-bounding it is also important to clarify that the human ‘STRANGEness’ I discuss here should not be confused with human ‘WEIRDness’ proposed by Henrich et al., in their article, “The Weirdest People in the World?,” (2010; see also Killin & Pain, 2023). In the latter case, weirdness indicates an epistemic problem that relates to how psychology and cognitive science studies (or rather disregards) cognitive variability. In particular, the problem is that major aspects of human psychology, as observed in the case of people living in Western, Educated, Industrialized, Rich, and Democratic (WEIRD) societies, differ from that of the rest of the world. As long as cognitive science continues to study participants mainly (if not exclusively) from WEIRD societies our knowledge will remain partial, wrongly assuming as universal, natural or even pathological, what is constructed and situated. You may think that philosophy lacking an experimental grounding can bypass this complication. This is not entirely true. For a big part of philosophy (perhaps the dominant part) is the product of WEIRD metaphysics. In this connection the recognition that people are STRANGE can provide the necessary unifying onto-epistemological foundation for practicing an anti-WEIRD philosophy by avoiding the debilitating pitfalls of relativism: If some people are WEIRD all people are STRANGE. This realisation provides the conditions of comparability necessary for revealing the astonishing diachronic and cross-cultural variability of human self-becoming (minimal and narrative) as this can be seen reflected in different aspects and phases of the archaeological and ethnographic record.

**Fig. 2:**
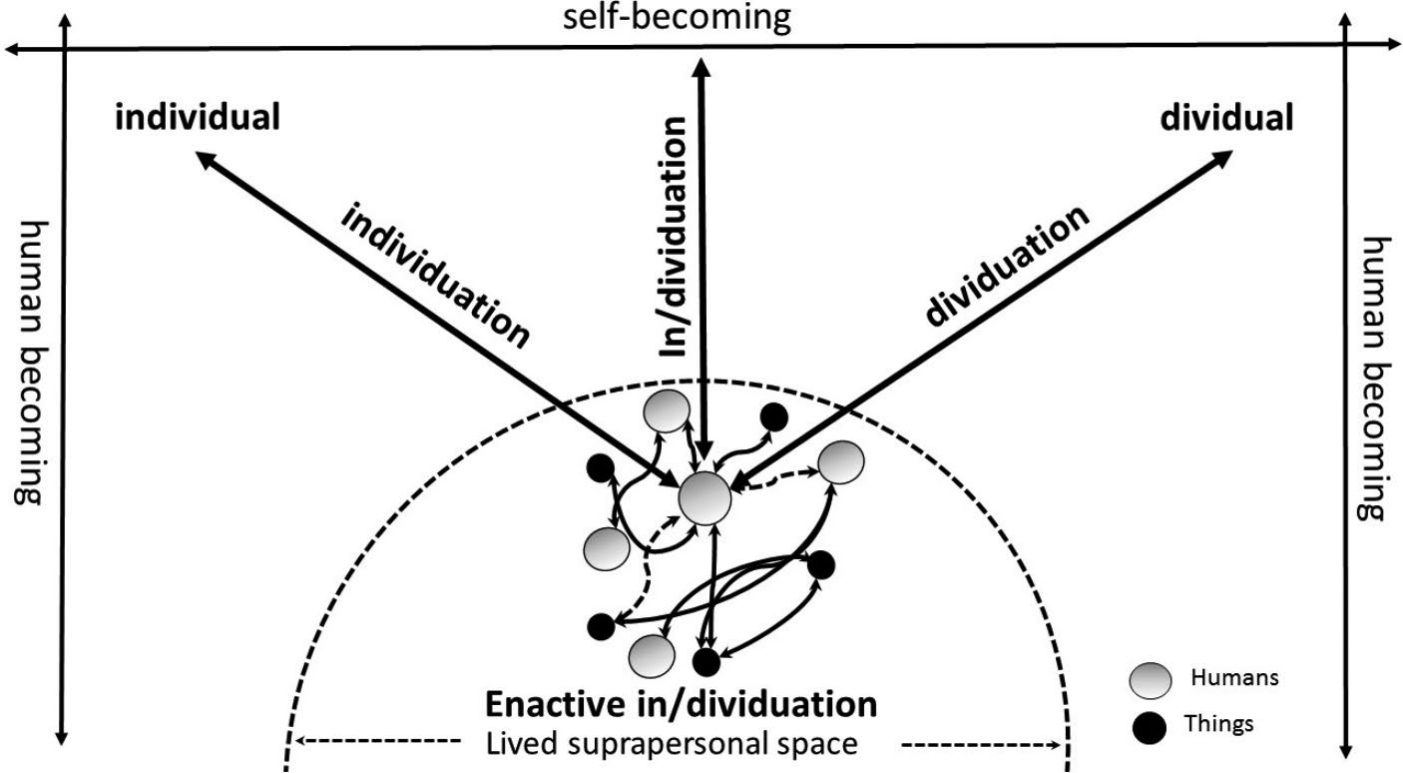
Enactive In/dividuation. Self constitution, seen as a process of enactive in/dividuation occupies a shifting position in the lived suprapersonal space. This shifting position (i.e., the situated person perspective SPP) is the dynamical product of the local entanglement between two processes: dividuation and individuation. Important to emphasise is that the states of dividuality and individuality co-exist as inseparable dialectical moments and should not be seen as separate antithetical kinds of self-becoming. Dividuality and individuality have no independent existence; they become constituted and can only exist as a mixture, i.e., the process of enactive in/dividuation. Important to note also is that the terms “individuation” and “in/dividuation” have different meanings. The first, “individuation”, denotes the conventional form of subjectivation by which autonomous differentiated selfhood is created. The second, “in/dividuation”, denotes the self-bounding processes by which the dependent situated selfhood is enacted. The predicate “enactive” indicates the grounding of in/dividuation on situated action and the primacy of material engagement

Accepting that people are STRANGE. We must also accept that it is only through the understanding of what being a self involves for an organism embedded inside a specific socio-material environment that one will be able to search, efficiently, for the constituents of human self-consciousness (neuronal, bodily, material or other). The problem of self is now recast as a *self-bounding* problem. The basic proposal that I want to put forward is that humans display a selfbound mode of becoming. The term selfbound (and the verb self-bounding) denotes the self-related processes by which human basic experiences and modes of material engagement are *bound* to an acting body in lived space to create and maintain self-continuity. Self-bounding is the process that allows self-knowledge to emerge by constantly blurring (making and unmaking) the boundaries between objects, persons and environments, while retaining an identity in the form of connections and entanglements that matter or else of self-patterns that connect (Bateson, 1973; see also Gallagher, 2024). My argument is that if human mind is unbound, extending beyond skin and skull, it is because human consciousness is selfbound. Specifically, I argue that *it is the bounding of consciousness that allows the unbounding of human thought and imagination*. Self-bounding is the precondition for a borderless mind. Selfbound is thought-unbound: it provides the material anchoring and self-grounding needed for the extension and distribution of human intelligence. Self-bounding denotes the process by which the degree of independence from, and relatedness to, the surrounding world is maintained or negotiated through discovering and reconfiguring one’s boundaries. The body matters for self not as an envelope, or delimiting boundary, but as an active, sensing and moving perspectival point for engaging the world. In short, the claim I put forward is that s*elf is less of a unitary entity inside a body and more of a bounding process of active exploration and sensorimotor engagement in suprapersonal space*. ‘Bounding’ here refers to the totality of enactive processes used in setting of the limits, constraints, or possibilities of in/dividuation. I call this process *enactive in/dividuation.* On this construal the mentioned anthropological division between ‘dividual’ and ‘individual’ personhood collapses. Instead of signifying antithetical poles in a dichotomy, ‘dividuality’ and ‘individuality’ now emerge as possible end points on a continuum of selfhood. Our exact position on this continuum varies and depends. There is no single position, or privileged point of view from where ‘I’ perceive the world. Instead, enactive in/dividuation signifies the ability of human consciousness to constantly integrate elements of ‘dividuality’ and ‘individuality’ by taking the perspective of self or the ‘other’, as well as by creating new technological and imaginary perspectives for experiencing the self and the world (Fig. 2).

The anthropologist Bird-David was the first to use the verbs ‘to dividuate’ or ‘individuate’ to express the sense of relatedness that characterise human subjectivity as a situated process. As she characteristically observe in the context of her examination of Nayaka’s modes of relatedness from South India (Bird-David, 1999):

“When I individuate a human being I am conscious of her ‘in herself’ (as a single separate entity); when I dividuate her I am conscious of how she relates with me. This is not to say that I am conscious of the relationship with her ‘in itself,’ as a thing. Rather, I am conscious of the *relatedness with* my interlocutor *as I engage with her*, attentive to what she does in relation to what I do, to how she talks and listens to me as I talk and listen to her, to what happens simultaneously and mutually to me, to her, to *us”* (1999, p. 72).

It should be noted that the process of enactive in/dividuation besides linked with Bird-David’s notion of ‘dividuation’, and the verb to ‘dividuate’, is also connected to the Gilbert Simondon’s conceptualisation of technical objects and the individual-milieu couple (1989 [1958]; 1992). The major assumption of Simondon’s philosophy is that the process individuation is biologically inseparable, but ontologically prior to the individual (or I will add to the dividual). The individual cannot be used as the point of initiation for understanding the process of individuation; rather, it is from the point of view of individuation that the individual can be understood (1992, 300). Specifically from the perspective of material engagement theory, the anthropologist Kåre Stokholm Poulsgaard (2019), in the context of his extensive ethnography with architects and engineers using digital computational design, coined the term ‘enactive individuation’—combining Simondon’s philosophy of individuation and Stiegler’s philosophy of technics^9^ with the enactive approach^10^—to describe how individual and milieu “emerge simultaneously from the tension inherent in individuation” by means of creative *thinging.* The archaeologists Fowler (2016) advancing a similar relational view of self has been addressing this tension between individual and dividual aspects of personhood proposing a multi-dimensional (rather than a single spectrum) approach according to which a series of dimensions may intersect with one another in varied ways. The in/dividual person then emerges as the product of this dialectical tension between the dividual and individual aspects of self that can be found to exist in all cultures albeit in different ratios, degrees of transparency, and ontological status (see also LiPuma, 1998). The exact dimensions and ratio of these tensions may differ both within and across communities of practice. Still the tensions are always there having an important creative role to play in terms of (a) how different varieties of self become enacted, mediated and distributed (or not) in time and space, and (b) where self-boundaries lie, how they become constituted, and when they shift.

It is here that the familiar pre-conceptual first-person perspective (1PP), proposed by phenomenologists, gives way to what we may call *situated person perspective* (SPP). In the first case, i.e. 1PP, the meaning of perspective is ‘directional’ where as in the latter, i.e., SPP, it is ‘transactional’ and ‘participatory’. Taking inspiration from the process philosophy of AN Whitehead the argument here is that self emerges (in evolution and development) not as the ‘subject’ of experience (from a first-person perspective) or the ‘object’ of reflection (from a third-person perspective), rather, it emerges as a transactional ‘superject’ (subject and object) (Whitehead [1929], 1978, 29). What that means is that humans experience subjectivity from a middle ‘in-between’ position. This position is not fixed but thoroughly embodied in situated and mediated action (self-becoming). The point of view of subjectivity is no longer ‘single’, ‘within/outside’, or ‘personal’; rather, it is ‘multiple’, ‘in-between’ and ‘suprapersonal’, i.e., the *situated person perspective*.

Being fundamentally a temporal and situated process, self-becoming is locationaly uncommitted. Obviously, such a decentralised conceptualisation changes the geography of the debate over the nature of selfhood. Our boundaries are shifting in response to our actions and the affordances of specific material environments. Self-boundaries can be expanded or contracted (e.g., to include or exclude the use of a tool). The collapse of those boundaries (as for instance in the case of immersion in skilful creative activity or in the case of self-fragmentation in extreme psychosis) would result in the ‘loss’ of self. By the same token, the objectification of boundaries, as when the Palaeolithic inhabitants of Blombos Cave in South Africa, that I discuss next, used shell beads and ochre to decorate their bodies, will intensify the self-other distinction.

## Self-semiosis in lived suprapersonal space

So where do we look for indexes of the self-bounding process in the material remains of the past? I suggest five domains of self-becoming that carry increased analytical potential. These are: (a) continuity in time, (b) agency (the sense that I am the one who is the initiator or source of the action), (c) ownership or *mineness* (the sense that I am the one who is undergoing an experience), (d) self-other distinction, and (e) the plasticity of self-boundaries. No doubt, the possible material traits of the above experiential domains are not easy to identify or isolate. In the last part of this paper I focus on two modes of bodily prostheses, well testified in the archaeological record, that can facilitate the study of the proposed domains. These are: tool making and using, and personal decoration. I will try to explain, what is that these practices can tell us about the emergence of self-exploration and self-objectification, and why I think that they can be seen as diagnostic of self-recognition, what we may call self-semiosis. I should clarify that the idea of self-semiosis should be differentiated from that of self-representation. Self-semiosis refers to the material or enactive signs (Malafouris, 2013, ch. 5) which different organisms may have access to, or engage with, in the course of their life history and which, one could hypothesize, can be associated with different aspects of self-identification. It is quite reasonable to assume that organisms who engage more frequently in behaviours that manifest or embody self-exploration, or self-objectification are more likely to develop self-awareness compared to those who do not. As I explain below, a stone tool and a perforated shell bead are material signs rich in self-semiosis which relate, nonetheless, to different aspects of human experience and intra-subjectivity.^11^ Different lithic production technics and modes of personal ornamentation allowed early humans to discover new ways of experiencing the body, its capabilities and its relation to the world as it converses and interacts with different materials within a changing field of affordances (interactive possibilities). That is, they provide cognitive ecologies that create new opportunities for self-bounding and enactive in/dividuation. The underlying assumption is that combined adaptations in these local ecologies of selfhood (creative and prosthetic) may have provided the necessary transactive material scaffolding (situated person perspective SPP) where self-other distinctions can be made, self-boundaries can be transformed, and new forms of ‘agency’ and ‘ownership’ can be (pre-reflectively) experienced and in some cases also (reflectively) conceptualised.

The working hypothesis here is that the material engagement practices of stone knapping and body decoration, each in their own ways, bring forth novel self-bounding environments that increase uncertainty in their respective hylonoetic fields and disrupt ordinary situated dynamics of bodily experience. The basic idea is that the early invention and development of knapping techniques and more recently of bodily decoration, by causing a rupture in established habits of engaging and knowing the world, effected a phenomenological ‘suspension’ which allowed early humans escape the taken-for-grantednes of their situation. This suspension, which is similar to what in phenomenology is known as *epoché* (the Greek word for ‘cessation’), provided access to new aspects of their bodily self and their relationship with their surrounding world that would have been otherwise impossible to achieve through normal ‘unmediated’ perception and bodily action.

Specifically, in the case of knapping (see section 4.1), I propose that the new entanglement between the hand, the stone, and the tool offered a novel tangible lived space for a deeper and extended phenomenological exploration of the actual experience of making. What I mean is that by learning how to make and use a tool, the knapper also learns new ways of experiencing oneself as an agent—learning here is understood both in the sense of enactive discovery we associate with improvisation and in the sense of social transmission. This type of self-related and self-motivated creative material engagement provides a new pathway into the consciousness of action: from pre-reflective consciousness *in* action (enactive intentionality) to conscious awareness *of* action. This consciousness of action is inseparable from feelings of ownership and agency that in turn provide the bodily foundation of self-consciousness. A similar kind of suspension of bodily self-experience can be seen also later on when we find the first evidence for personal decoration (section 4.2). *Epoché* here consists in the shift of attention from the way bodies appear, from the subjective first person point view to the way they are constituted by consciousness of the ‘other’ i.e., the body as it is being observed by others. Bodily decoration is liberating the self from the temporal simultaneity and spatial coincidence of the subjective body so it can be fully enchained and embedded into its social surrounding (Malafouris, 2008a,b).

### Tools, self and the body

It is widely recognized that the emergence of intentionally modified stone tools marks a significant step in human evolution (e.g. Shea, 2013; Wynn, 2002; Stout, 2011; Wynn, 2021). Starting with the earliest simple, Oldowan (Mode1) lithic assemblages, to the more standardized bifacial Acheulean (Mode 2) technologies that emerged around 1.7–1.5 Ma, to the Levallois (Mode 3), and last, to the blade-based microlithic technologies of the Upper Palaeolithic (Mode 4–5) (Ambrose, 2001; Foley & Lahr, 2003) a chief concern has been to record and understand the changes in terms of reduction sequences and perceived degree of technological competency. Could the observed diversity in lithic assemblages and in the technical competency of tool makers as this can be studied by looking at core reduction sequences and comparing the ways hominin knappers displayed sequential planning and an understanding of core geometry and properties of stone (e.g. Roux & Bril, 2005) inform us also about possible transformations in self-consciousness?

Let us return to the example of the early tool maker from the Olduvai Gorge in Africa preparing a sharp-edged tool with which I started this article. I propose that the production of Oldowan stone flakes by means of percussion represents a minimal form of self-bounding and one of the earliest *techniques of the self*. Percussion—broadly defined here as the controlled elementary action of using an object (a ‘hammerstone’) to strike the surface of another object (the stone ‘core’) (see Whiten et al., 2009)—is the most basic technique of stone tool making. It sounds simple. However, the complexity of percussive movement is greater than one might think. Successful striking actions demand bodily coordination, control of manual grips, and fine perceptual-motor skills (Roux & Bril 2005; Nonaka et al., 2010). The Oldowan hominin repertoire of stone flaking techniques is an exercise in the coordination, precision and timing of action and bodily movement. It is also an actualisation of intent. We should not be thinking of this actualisation as the externalisation of pre-conceived intentional states through a pre-ordered operational sequence of technical gestures. Knapping movements are always situated; they occur in context and are inseparable from their material environment. They are sentient movements that remember their past by leaving their traces on the rock’s surface. They also project into their future, anticipating and predicting the position of the next strike as well as the sharpness of a cutting edge they aim to produce (Malafouris, 2021a,b; Ihde & Malafouris, 2019).

To understand what it means to produce an intention-to-knap-stone, that is, to understand the meaning of ‘intentionality’ in early human toolmaking, it is important to understand first the different kinds of operative intentionality involved (e.g. intentions prior to flaking and enactive intentions in flaking) and their possible links with processes of attentive and predictive material engagement. Such an understanding is inseparable from the temporal structure of the knapping experience. By temporal structure I refer specifically to the phenomenological integration of past, present and future into an “intentional arc”^12^ consisting of a “retentional”, “presentational” and “protentional” function (Husserl, 1964) of the experience of knapping. Knapping is often presented and modelled as a linear operational sequence (the unfolding of a pre-determined action script), but from a material engagement perspective it should be better understood as dynamical, multi-temporal (non-linear) and distributed process of *thinging* (Malafouris, 2021a,b). The flaking act is both the cause and the consequence of the flaking intention.

**Fig. 3:**
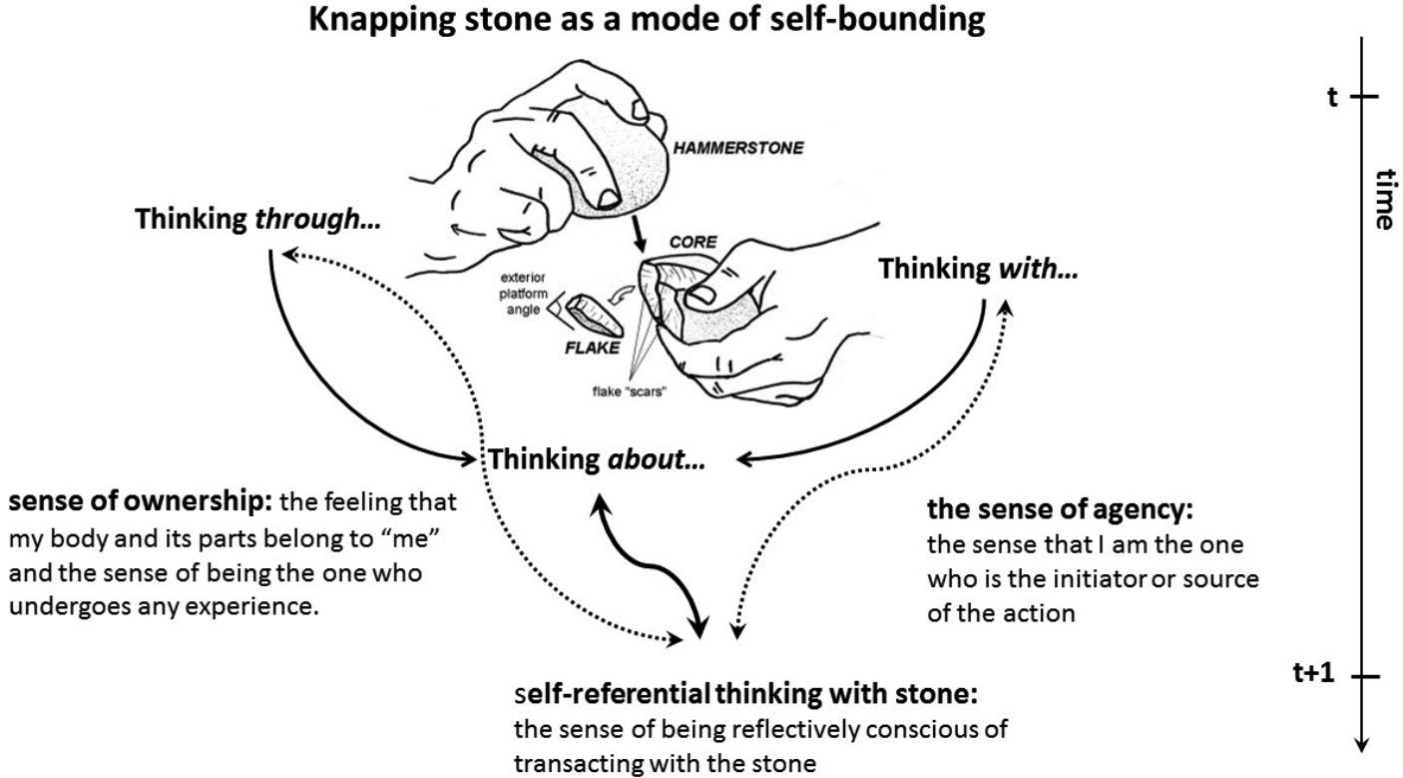
Stone knapping and self-exploration. The practice of knapping stone provided the tangible physical space for self-exploration through the experience of making. The attentive and creative engagement between hand and tool facilitated the emergence of minimal self-knowledge (sense of agency and ownership of the ‘subjectively felt’ and ‘objectively seen’ body). Tool making opens up a new pathway into the consciousness of action: from pre-reflective consciousness in action (enactive intentionality) to conscious awareness of action. From a MET perspective the underlying hypothesis is that the knapper first thinks through and with the stone before being able to think about the stone as a conscious and reflectively aware agent. In other words, the situated person perspective (the knapper’s thinging) provides the cognitive ecology needed for enactive in/dividuation

One important question here is whether some kind of minimal self-knowledge is required to be able to have an intention-to-knap-stone; or conversely, whether self-knowledge may be produced as a result of the knapping activity? I suggest that tool making and using, at least at the level observed in early prehistory, does not require self-awareness. The reason I propose that the origins of self-experience can be intertwined with percussion is not because I think that flake-making (the ability to consciously control the shape of a flake) demands or presupposes the existence of a self-aware individual maker capable of entertaining intentions, exerting executive control and attention. Rather, I propose that self-experience and percussion are entangled because percussion instantiates a field of hylonoetic activity that enables the transformation of unbounded energies (neural, bodily, material) into selfbounded agencies. This transformation is made possible through the production of material form (for instance, an edge or a cutting tool). In other words, I suggest that a basic self (comprising of a sense of agency and body ownership) is not a requirement for knapping; rather, it should be seen as the product of the knapping process or, in any case, as something experienced during acts of knapping (Fig. 3).

Applying the principles of MET and the logic of self-bounding the underlying hypothesis is that the knapper first thinks *through* and *with* the stone before being able to think *about* the stone as a conscious and reflectively aware agent. In other words, the situated person perspective (the knapper’s *thinging*) provides the cognitive ecology needed for enactive in/dividuation. The entanglement of gesture and tool brings forth the *tectonoetic* awareness (from the Greek *tecton* signifying the maker and the poetic art of construction and the word *noϋs* for mind or intellect) needed for the developmental passage from noetic to *autonoetic* consciousness (from the Greek *auto-* for self- and *noêsis* for intelligence)^13^ (see also Malafouris, 2008a).

Learning and practicing the art of knapping the early knapper develops a new understanding of the complex ways the body can be responsive to one’s will, and attribute the source of action to oneself. The knapper also learns to distinguish actions that are self-generated from those that are not, as well as that ‘others’ are potentially agents of their actions. At a basic level, knapping provides information specifying the self as distinct from the world on the basis of the demands it places on one’s direction of hand movement and control of posture. Tactility and the human hand play a unique role in realising our agentic capacities during visuospatial integration and eye-hand-tool coordination (Bruner et al., 2019; Bruner, 2023). We should not forget that the hand is the part of our body that we engage visually the most and that modulates perception and human-world interaction. Importantly, the hand is the most creative part of the human body and the principle interface of the entanglement between early humans and their material environment. This multimodal specification of basic self is embedded in action and entangled with the specific spatio-temporal parameters of the tool-making tasks. The dynamical parameters that specify the topology of the stone-knapping task (e.g. point of percussion, angle of blow and exterior platform angle) specify also the changing topology of self-location (personal, peripersonal or extrapersonal). We could test that by looking at actualistic studies and collecting data from replicative experiments (behavioral & neuroimaging) conducted with modern stone knappers with different skill levels (e.g. Stout et al., 2008; Stout & Chaminade, 2007; Shea, 2015; Pargeter et al., 2019, 2020).

With time and practice the toolmakers notice the consequences of their actions and learn how to effectively situate their body and use their hands and available resources in order to maintain desired effects. They learn to touch by seeing and to see by touching. Moreover, learning to anticipate outcomes of their actions they also develop the ability to think and project into the future. The accumulated embodied knowledge allows the knapper to predict the consequences of a strike given to a core. That way, the knapper acquires a feeling of control over its bodily movement. It makes good sense to hypothesise that there must be a strong link between the ability to predict and control the shape of a flake (successfully or not) and the sense of agency. Important to repeat here is that, when I am saying that in making tools hominins also learn to imagine and predict the future consequences of their actions, no priority of mentality over physicality is implied. From an archaeological perspective there is no reason to assume that an internal ‘brainbound’ approach to cognition offers the natural starting point for understanding the processes involved in building up self-consciousness. The predictions I discuss here are situated: they denote the kind of predictive engagement referring to “a dynamical adjustment” or attunement by which the organism (brain and body) actively responds and interacts with the changing socio-material environment. In short, the situated knapper (brain and body) pre-reflectively anticipates the shape of a flake in a multi-temporal manner that combines protention, attention, and intention. This process although not incompatible should not to be confused or reduced to the kind of neurocentric *predictive coding* associated with internal Bayesian models and prediction error minimization (Gallagher & Allen, 2018; Gallagher, 2023b; Clark, 2024).^14^

What allows the early toolmakers to project in time and think about themselves into the future, is as much the product of neural-based anticipation (realised in the head) as it is of extra-neural material imagination (realised in the world on the stone’s surface). Within such multimodal processes of creative material engagement, it is especially hard to maintain the old distinctions between the domains of perception, cognition and action. Similarly, there are no fixed agentive roles in this process. The same difficulty applies for the boundaries between personal and peripersonal space. Those must change too to accommodate the incorporation of tools (Malafouris, 2013). This applies whether the tool is perceived as an extension of the hand, assimilated into the body schema, or as a powerful means for objectifying the hand as a tool (Malafouris, 2008c). The body that uses and incorporates that tool is a different kind of body than the one who does not. It is a different body because the sense of what it can or cannot do or experience has changed. As such, toolmaking practices and skills by reaching deep into the supposedly immune compartments of our minimal or basic self and embodiment may have provided the necessary scaffolding for the emergence of self-consciousness and the experience of agency.

### Beads, self and the body

Early body decoration is the second major domain for our analysis. The body’s surface seen as “a cultural palette for decoration and modification” (Farnell, 2011, p. 141) has long been subject to anthropological and archaeological attention relevant to issues of cultural inscription and the symbolic construction of personal identity. Personal decoration is also generally accepted as one of the earliest archaeological expressions of modern cognitive abilities loosely defined in terms of representational abilities (e.g. Henshilwood et al., 2004). Archaeological discoveries such as the shell beads recovered at the Blombos Cave, which date at c. 75 kya (d’Errico et al., 2005) or those from Grotte des Pigeons in Morocco, which date at c. 82 kya (Bouzouggar et al., 2007) have led many archaeologists to see in these objects an unambiguous marker of symbolically mediated behaviour and, by implication, of language. However, an argument can be made that the precise inferential steps that allow archaeologists to ascribe symbolic intent to these objects remains elusive. I have proposed self-awareness as a useful alternative to rethink those issues (Malafouris, 2008b; Iliopoulos & Malafouris, 2021). In particular, my suggestion is that a more productive way to approach the issue of early personal decoration is to think of it along the lines of enactive signification (rather than abstract symbolic capacity) and to explore the role it may have played in transforming the phenomenological self-as-subject to a social self-as-object. Rethinking the evidence for early bodily ornamentation on such an enactive foundation, and contextualising that evidence relevant to other material and technological innovations from the same periods (see Henshilwood & Dubreuil, 2011), we can hypothesise that personal decoration made possible the bringing forth of a new type of reflective self-knowledge. This should not be understood to imply that only shell beads or body decoration could have played that role. As discussed previously, tool making practices, had already facilitated the emergence of a basic self by objectifying the sense of agency and body ownership which are essential for the development of human autonoetic awareness. The proposal is, nonetheless, that early body-decoration brings some additional situational affordances that allowed the development of self-experiences and modes of self-bounding that we don’t see in the case of tool use and manufacture. These situational affordances of bodily ornaments and their special epistemic qualities relate to the capacity of the beads, as material signs attached to the body, to make visible and tangible what is inherently silent and transparent: the phenomenological distinction between the body-as-subject or “Leib” (i.e. the pre-reflective “I”) and the body-as-object or “Korper” (i.e. the physical body that is observable both by myself and by others) (Merleau-Ponty, 1945[1962]).

Beads as any other form of body decoration are always for-someone; they involve an experiencer (individual or collective). What maybe is even more important from a material engagement perspective, is that they can be associated with a new ‘perspectival’ sense of ownership: the use of the shell-beads provides a non-linguistic reflexive pronoun referring back to the body concerned. It offers a new way of ‘pointing’ to the individual body in question. Seen as a material sign the shell bead objectified personal ownership and the quality of ‘mineness’ in a way that no other artefact has ever done in early human prehistory raising important questions concerning the relation and distinction between self and other.

The bead attached to the body plays a role analogous to that of possessive prefixing terms and personal pronouns, that is, it acts as a predicate of oneself. The beads provide a material anchor for a process that both recognizes, relates and separates self and other. This process is not symbolic or representational but affective, enactive and indexical. One way to analyse this process is in the form of a self-other relation/differentiation: personal ornaments afford exchange, partibility and in/dividuality. I do not see this exchange as part of an explicit communicative symbolic code but as a process primarily enacted as a kind of gestural/bodily language. The key element here is not symbolic capacity, or the ability of mentalising (reading each other’s minds), but ‘attachment’. To describe these relations of attachment that characterise body decoration (paint, tattooing, piercing and other modifications), some anthropologists have used the term “second skin” (Turner, 1980). In this sense the shell bead attached to the Palaeolithic body operates as a visible, corporeal extension of the skin. The anthropologists Alfred Gell in his work on tattooing in Polynesia gives us a good example of this process. He describes tattooing as an exteriorisation of the interior “which is simultaneously the interiorisation of the exterior” (1993, 38 − 9). With tattoo, Gell wrote, “the body multiplies; additional organs and subsidiary selves are created” (1993, 39; see also Ostojic & Taylor, 2023). Adding this brief anthropological note in our discussion I want to highlight two things: first that there is clearly a relationship between self and the surface of the body that differs in different cultural contexts. Second, that the sociomaterial relations embodied in terms such as ‘second’ or ‘social’ skin (Turner, 1980) cross-cut the conventional ‘body image’/‘body schema’ distinctions (Gallagher, 2000; Malafouris, 2008c) and thus, should not be reduced to some symbolic or inscriptive ‘disembodied’ realm.

The bodily ornament, like a tool, can be seen as an extension of the body and thus a part of the self. However, whereas the making and use of a stone tool can be seen primarily as a *creative and prosthetic gesture*, personal decoration can be primarily described as *a pointing gesture*. Of course, in the case of bodily decoration it is not simply the hand that is gesturing but rather the whole body or whatever parts of that body one chooses to decorate. This also means that in contrast to what we have been discussing in the case of tool manufacture, it is not the manual dexterity and precision of the knapper’s hand that provides the locus of agency, but the mere appearance of the decorated body. Decoration offers a new prosthetic means by *which the bodily-self gestures towards the other*. In that sense, the shell bead provides a bodily technique for revealing or disclosing the self. This function is not symbolic or re-presentational but inherently enactive or presentational. The ornament acts as an artificial ‘fingering’, a device for enactive discovery and of self–other differentiation by ‘pointing out’ the self and the other. Seen as a pointing gesture bodily decoration provides an early example of the distinctively human form of consciousness that Raymond Tallis calls “indexical awareness”. As he explains in *Michelangelo’s Finger*: “[t]his is a form of awareness that, as it were, points to itself, or points to its own source. The awareness points back to the one who is aware. I am a touched toucher, a seen seer. In short, I am present to *myself* in the field of things that are present to me. I am next to the object and the object is next to me” (2010, 28, original emphasis). In particular, attached to the body the bead becomes a visible, corporeal extension of the skin and modification of that body. At the same time, seen as a dual entity that both touches the body and faces outward toward the other, the shell bead can be described as an artificial skin capable to reshape the body, to which it becomes attached (extending near ‘peripersonal space’).

I argue that body decoration is capable of liberating the self from the bounds of the bodily self and the personal space so it can now be enchained into its social surrounding in peripersonal and extraperonal space (Malafouris, 2008b). However, I also believe that the key element or property behind this social enchainment is not the symbolic communication of social identity, such as group membership, gender etc., that many archaeologists have proposed drawing parallels with the function of personal decoration in recent human societies. As discussed, the presence of reflexive self-awareness and self-other distinction should not be taken for granted. The key element or property here is not ‘symbolism’ but ‘engagement’. The shell bead is important for what it *does* and not for what it *means*. Early body ornamentation like what we see at Blombos Cave during the MSA should not be understood as existing for representing the self-other distinction; rather, we should be seeing the self-other distinction as enacted through the ornament (see also Garofoli, 2015; Garofoli & Iliopoulos, 2019).

I do not wish to question the basic intuition that the presence of personal decoration indicates the developing awareness that humans live in a world of other minds (human and nonhuman) and that they think of their selves with *others in mind* (Rochat, 2009). I want simply to emphasise the active role that bodily ornamentation may have played in mediating and constituting intrapersonal subjectivity. More simply, instead of seeing in early ornaments a symbolic means by which the transactable person signifies social distinction or identity by decorating the body, we should be seeing the social self (the second person perspective) as emerging through skilful transaction actively mediated by the ornament. The development of self–other awareness is partly constituted by the affective capacity of the ornament. The shell bead attached to the body does more than merely symbolise group identity or personal identity as part of a group. Rather, it provides the actual material means for such a ‘togetherness’ to be performed, incorporated and inscribed in a manner and form that uninscribed bodies can hardly attain. In short, biosocial assemblages of beads and bodies transact in intrapersonal space to bring about a new performative understanding of ‘togetherness’ and ‘we’ or ‘joint’ intentionality. This new understanding of intersubjectivity and ‘we’ intentionality is one that is no longer based on the disembodied interactions between human individuals (as for instance discussed by Tomasello et al., 2005) but now includes non human social actors and material culture in equal terms. The emergence of explicit self-other distinction presupposes that in some minimal sense ‘the other’ is revealed through bodily presence and movement. This bodily mode of presentation also reveals subjectivity capable of mental acts and states (thoughts, emotions etc.). It is especially this kind of empathy that the ornament enhances and accentuates towards understanding others’ perspectives. However, the way to interpret that is not as a kind of inferential simulation process associated with ‘Theory of Mind’ but in the enactive sense of in/dividuation, where dividuality is no longer opposed to or contrasted with individuality; rather the two concepts unite to describe the co-constituents of self-becoming. Social cognition and the intersubjective sense of self as dependent on the perspective of others is more than a passive metalizing of the other. Personal decoration seen as an enactive mode of self-semiosis enables not just a social understanding *about* the other but also an experience or feeling of self-bounding *with* the other. However, this social understanding presupposes nothing more but a pragmatic ability or non-verbal know-how of acting together and forming appropriate relations in specific situations.

## Epilogue

The general idea guiding this article is that, humans (person and species) continuously evolve (become) as relational (constitutively open) sentient creatures undergoing situated ontogenetic histories. And yet, against this background of constant perturbation and transformation humans are capable of enacting a mode of autonoetic existence. That is, they display a sense of self that persists in time, offering the semblance of identity and continuity that we have (or maybe the illusion of it). If self-becoming is central to realizing one’s humanity (social, moral, technical, linguistic, political or other), what is that self made of, how exactly it comes about and what possible forms does it take?

Those general questions run through this article and I have tried to address them within a theoretical framework of material engagement (Malafouris, 2013, 2019) focusing on a few selective but representative archaeological themes. My ambition has been to recommend a new direction of thinking, combining philosophical and archaeological perspectives, that can help us to rethink (also to unthink) the major processes and forces that bind humans to one’s self. The theoretical upshot of this paper is that, rather than conceiving of self-consciousness as ‘internal’ and independent from the material world, it is entangled and fundamentally co-dependent with it. Adopting the material engagement approach, and combining enactive, embodied and ecological trends in philosophy of mind and cognitive archaeology I have tried to excavate the multi-temporal stratigraphy and reconstruct the suprapersonal topology of the middle territory of enactive in/dividuation where self-bounding happens.

Approaching the making of human self-consciousness as a ‘middle’ problem, beyond nature and culture, brings about the need for better epistemic unification among the different aspects of self-becoming as they interact at the intersection of mind and matter and across the scales of time. Philosophical and cognitive archaeology opens up a possible way to do so by (a) identifying in the archaeological record different phases or trends of radical reconfigurations of human peripersonal space, (b) constructing a long term temporal statigraphy of these plastic transformations, and (c) comparing them with other marked transitions of phases in human cognitive evolution. The insights gained from such a study might help us rethink the traits that mark the origin of our species and advance the ongoing debate on human becoming by dissolving some of the confusions surrounding the ontology and evolutionary significance of early modes of material engagement. Comparing different material forms and techniques of self-bounding, from early prehistory to the present, can lead us to a more critical view on the meaning of stability and change in human beings and the possible shape of future human becoming.

## Data Availability

Not applicable.

## Footnotes

1 Husserl describes this complication as the “paradox of human subjectivity: being a subject for the world and at the same time being an object in the world” (1970, p. 178).

2 Mereology (from the Greek *meros* ‘part’) refers to study of the relations of part to whole and the relations of part to part within a whole (see De Vignemont et al., 2006).

3 The notion of ‘dividuality’ was introduced to explain the differences between the relationally constituted non-Western (specifically Melanesian) person who may be divided and distributed among objects circulating through exchange from the Western autonomous, self-animated, and self-enclosed agent (Strathern, 1988).

4 As Charles Taylor remarks in *Sources of the Self* not only do we feel ourselves as firmly located within us but we also feel we have selves in the same way that we have heads, legs, arms or livers: “distinctions of locale, like inside and outside, seem to be discovered like facts about ourselves, and not relative to the particular way, among other possible ways, we construe ourselves” (1989, 112).

5 Dumont (1999) has in fact traced the origins of the concept of ‘individuality’ as the bounding condition of selfhood to the work of the early Christian thinkers and in particular St. Thomas Aquinas.

6 Here I am following John Dewey’s conceptualisation of situation as constituted by the organism-environment (1938). See also discussion in (Gallagher, 2014, pp. 115-6).

7 I borrow the term from the philosopher Evan Thompson. As he points out from a similar enactivist perspective: “the crucial ingredient that takes us from a self-specifying system to a full-fledged I-making system is that of being ‘self-designating’ system. A self-designating system is one that can designate itself as a self. This means it can attend to its changing experiential states and conceive of itself as the subject of those states” (2014, 344.)

8 Stiegler also coins the term “epiphylogenetic memory” to denote the mode of tertiary memory by which past epigenetic process come to be accumulated within the spatio-temporal organisation of material environments (1998).

9 Esp. his concepts of *grammatization* and *epiphylogenetic memory* (see Stiegler, 1998).

10 For other recent attempts at combining enactivism with Simondon’s philosophy see also (Dereclenne, 2021; Di Paolo, 2020).

11 The same variation, of course, is to be expected even within a single category of evidence. For instance, the actions taken to control the shape of a flake through direct hard-hammer percussion and those involved in the making of compound, heat-treated glues 70,000 years ago (Wadley, 2010) provide different affordances for attentive material engagement and personal continuity (Wynn, 2021).

12 Merleau-Ponty coined this term to denote the tight connection between the agent and the world brought about by means of perceptive and motor schemas allowing for a basic or ‘operative intentionality’ (1962).

13 The terms *noetic* and *autonoetic* awareness were initially proposed by Tulving (1983, 2002). In particular, *noetic* awareness refers to the basic sense of oneself as acting in and on the environment at a time according to one’s first person perspective (1PP). *Autonoetic* awareness broadly refers to the sense of oneself as a unique individual persisting over time able not only to interact with objects and others according to one’s first person perspective (1PP) but also to reflect on one’s perspective. *Noetic* consciousness is knowing, *autonoetic* consciousness is self-knowing.

14 As Gallagher and Allen explain (2018): “perception and active inference are two sides of the same precision-weighted process allowing for the prioritization of actions that are likely to produce predicted outcomes and the contextualization of perception by prior action and history. This dependency of action on the structure of perception (and vice versa) provides a deeply embodied form of engagement, where the priors and actions an organism is likely to entertain are fundamentally constrained and afforded by the morphological structure of the agent’s body. In this way, the Bayesian brain is uniquely equipped to exploit the finely tuned properties of an organism’s dynamic morphological body and associated *Umwelt.”*
